# Multi-omics investigation of high-transglutaminase production mechanisms in *Streptomyces mobaraensis* and co-culture-enhanced fermentation strategies

**DOI:** 10.3389/fmicb.2025.1525673

**Published:** 2025-02-05

**Authors:** Huanan Chang, Ziyu Zheng, Hao Li, Yanqiu Xu, Gengyao Zhen, Yao Zhang, Xidong Ren, Xinli Liu, Deqiang Zhu

**Affiliations:** ^1^Shandong Provincial Key Laboratory of Microbial Engineering, School of Bioengineering, Qilu University of Technology, Shandong Academy of Sciences, Jinan, China; ^2^State Key Laboratory of Biobased Material and Green Papermaking, Qilu University of Technology, Shandong Academy of Sciences, Jinan, China

**Keywords:** transglutaminase, complex mutations, genomics, transcriptomics, co-culture fermentation

## Abstract

Transglutaminase (TGase) has been widely applied in the food industry. However, achieving high-yield TGase production remains a challenge, limiting its broader industrial application. In this study, a high-yield strain with stable genetic traits was obtained through UV-ARTP combined mutagenesis, achieving a maximum TGase activity of 13.77 U/mL, representing a 92.43% increase. Using this strain as a forward mutation gene pool, comparative genomic research identified 95 mutated genes, which were mostly due to base substitutions that led to changes in codon usage preference. Transcriptomic analysis revealed significant expression changes in 470 genes, with 232 upregulated and 238 downregulated genes. By investigating potential key regulatory factors, comprehensive analysis indicated that changes in codon usage preference, amino acid metabolism, carbon metabolism, protein export processes, TGase activation, and spore production pathways collectively contributed to the enhancement of TGase activity. Subsequently, the in vitro activation efficiency of TGase was further improved using co-cultivation techniques with neutral proteases secreted by *Bacillus amyloliquefaciens* CICC10888, and a TGase activity of 16.91 U/mL was achieved, accounting for a 22.71% increase. This study provides a comprehensive understanding of the mechanisms underlying high-yield TGase production and valuable insights and data references for future research.

## 1 Introduction

Transglutaminase (TGase, EC 2.3.2.13) catalyzes the formation of cross-links between the γ-carboxamide group of glutamine residues and the ε-amino group of lysine residues in proteins, as well as deamination reactions with water (Yokoyama et al., 2004). This cross-linking is particularly effective in food proteins (Duarte L. et al., 2020), allowing TGase to bind minced meat into cutlets while preserving sensory properties such as taste, texture, and appearance (Hong et al., 2016; Lesiow et al., 2017). TGase can also enhance the nutritional value of meat products by using raw materials like collagen and mechanically deboned meat, supplementing them with essential amino acids like lysine (Kieliszek and Misiewicz, 2014). In addition, TGase is applied in the production of dairy products (Færgemand and Qvist, 1997), where casein and whey proteins in milk serve as good amide donor and amino donor substrates for TGase, respectively (Oner et al., 2008). The use of TGase enables the production of protein-based film materials instead of plastic packaging, which used for fresh fruits and vegetables to improve shelf life (Marquez et al., 2017). These protein-based film materials are non-toxic, edible, and biodegradable, and they have good mechanical strength and excellent air permeability (Porta et al., 2016). Consequently, TGase has a wide range of applications in the food industry, including meat products, dairy products, cereal products, soy products, and food packaging.

**FIGURE 1:**
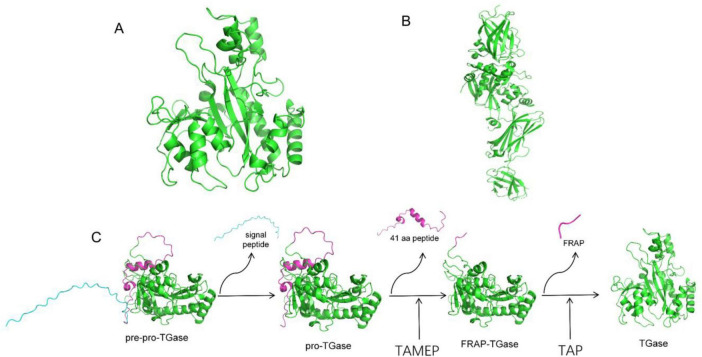
Simulated three-dimensional structure of TGase. **(A)** Three-dimensional protein structure of *S. mobaraensis* TGase. **(B)** Three-dimensional protein structure of human TGase. **(C)** pro-TGase activation process. The TGase in the intracellular signal peptide of the precursor protein recognizes and cleaves the signal peptide, translocates to the extracellular space in the form of the precursor protein of pro-TGase, and then excises the first 41 amino acids of the enzymatic proximal region by TAMEP. The first 41 amino acids of the pro-TGase region generate FRAP TGase, and then TAP excises FRAP to generate TGase.

TGases are widely distributed in nature, and they can be classified into two main categories: animal-derived TGases and microbial TGases. Studies have shown that TGase is widely present in animal tissues and body fluids, playing roles in various physiological processes, such as blood coagulation, skin keratinization, erythrocyte membrane stabilization, and wound healing (Aeschlimann and Paulsson, 1994). However, its biological function in microorganisms remains incompletely understood. This enzyme is known to associate with cell walls and may participate in covalent protein cross-linking, including the formation of cross-links between cell wall proteins (observed in *Streptomyces*, *Candida albicans*, and *Saccharomyces cerevisiae*) and in spore coat proteins (in Bacillus species) (Duarte et al., 2020). Although the TGase gene is non-essential for bacterial growth, its knockout leads to the formation of non-sporulating or bald colonies in S. hygroscopicus and impairs bacterial adaptability to harsh environments (Chen et al., 2013). Since 1989, Ando et al. (Ando et al., 1989) have been screening a *Streptomyces mobaraensis* species that can secrete TGase. At present, this species is widely studied and used in industrial production. It was later discovered that certain strains within the *Bacillus genus*, such as *Bacillus circulans* (de Barros Soares et al., 2003) and *Bacillus subtilis* (Liu et al., 2018), can also produce TGase. However, their production levels are relatively low. TGases of Streptomyces origin are different from those of mammalian origin, with little sequence similarity (Figures 1A,B; Oteng-Pabi and Keillor, 2013). In addition, TGase of Streptomyces origin do not require calcium ions to maintain their activity compared with those of mammalian origin, and substrates are broadly diverse with relatively small molecular weights, which indicate their low cost and wide industrial applications (Yokoyama et al., 2004).

The synthesis and activation of TGase in *S. mobaraensis* are complex processes involving multiple enzymes (Figure 1C), and as research continues to deepen, the relationship between this process and related biological processes also has a non-negligible impact on the efficient synthesis of TGase, with a single gene overexpression often resulting in less than the expected research benefit. Sustainably increasing the fermentation activity of TGase has been a hot topic for decades. Yin et al. (2021) applied atmospheric and room-temperature plasma (ARTP) mutagenesis to smWT and successfully obtained a high-yield TGase strain, with a transglutaminase activity that was 5.5 times higher than that of the original smWT strain. According to Zhang et al. (2011, 2019), the enhanced TGase production in *S. mobaraensis*, which was achieved by excessive MgCl_2_ treatment, reached 4.3 U/mL at 96 h of fermentation. A subsequent proteomic comparative analysis revealed that MgCl_2_ stress enhanced the expression of proteins involved in energy metabolism, stress response proteins, and enzymes associated with the synthesis of limiting amino acids, thereby supporting the synthesis of Xavier et al. (2017) optimized the nutritional components of the culture medium and cultivation conditions (including pH, temperature, agitation speed, and fermentation time), achieving a TGase activity of 4.1 AU/mL. Nagy and Szakacs (2008) selected different substrates for the production of TGase and optimized the TGase activity up to 5.1 IU/g by performing solid-state fermentation on different substrates. Yang et al. (2009) expressed pro-MTG-His6 in high-density cultured Escherichia coli BL21 (DE3) cells and achieved a maximum TGase activity of 8 U/mL after 6 h of induction. Although applicable gene editing and gene expression enhancement molecular biology tools for *S. mobaraensis* have been developed in recent years, Fang et al. (Yuan et al., 2024a) employed several engineering strategies, including promoter engineering, signal peptide optimization, enhancement of recombinant sites, and increasing the copy number of expression cassettes. These approaches collectively increased the enzyme activity to 63.18 U/mL. Ye et al. (2024) investigated the impact of specific genes on enhancing TGase activity in *S. mobaraensis* using gene editing and gene expression enhancement techniques. Based on their results, by combining random mutagenesis with site-specific gene integration, a thermostable TGase mutant, TGm2, can be efficiently synthesized in *S. mobaraensis*, achieving an extracellular TGase activity of 61.7 U/mL. However, this finding still needs improvement compared with the research results of protein synthesis in other strains. Given the limited understanding of the metabolic regulation mechanism of *S. mobaraensis* TGase, the impact of molecular modifications in *S. mobaraensis* has been far less than expected. Therefore, in-depth analysis of the TGase synthesis, the modification and secretion of high-yielding bacteria using traditional methods, and the excavation of unknown key control factors is necessary to guide subsequent experiments.

The aim of this study is to address these limitations by comprehensively analyzing the genetic and transcriptomic changes associated with enhanced TGase production in mutant strains of *S. mobaraensis*. Using comparative genomics and transcriptomics, we seek to identify key genetic factors contributing to high TGase yield and validate their expression through quantitative real-time PCR (qRT-PCR). Additionally, we employed a novel co-cultivation strategy with Bacillus amyloliquefaciens, whose secreted neutral proteases significantly improve TGase activation. This approach, not previously explored in TGase production optimization, offers a practical solution to longstanding challenges in fermentation process enhancement. The findings provide valuable insights into the regulatory mechanisms underlying TGase biosynthesis and suggest strategies for further improving industrial enzyme production.

## 2 Materials and methods

### 2.1 Strains and culture environment

Strains: The starting strain SmDL is kept in our laboratory. *Bacillus amyloliquefaciens* CICC10888 was purchased from China National Research Institute of Food & Fermentation Industries Co., Ltd.

Culture media: Gauze’s No. 1 medium (soluble starch 20 g/L, KNO_3_ 1 g/L, K_2_HPO_4_ 0.5 g/L, MgSO_4_⋅7H_2_O 0.5 g/L, NaCl 0.5 g/L, FeSO_4_⋅7H_2_O 0.01 g/L, and agar 20 g/L; pH 7.4–7.6). Seed medium (glucose 20 g/L, peptone 20 g/L, yeast extract 5 g/L, MgSO_4_⋅7H_2_O 2 g/L, K_2_HPO_4_ 2 g/L, and KH_2_PO_4_ 2 g/L; pH 7.0). Fermentation medium (glycerol 20 g/L, peptone 20 g/L, yeast extract 5 g/L, corn steep powder 20 g/L, KH_2_PO_4_ 4 g/L, K_2_HPO_4_ 2 g/L, and MgSO_4_⋅7H_2_O 2 g/L; pH 7.0). 2 × YT (peptone 16 g/L, yeast extract 10 g/L, and NaCl 5 g/L) was used for spore germination.

Cultivation conditions: Both the wild-type and mutant strains were cultured on Gauze’s No. 1 medium at 30°C for 6 days to prepare a spore suspension with a concentration of about 10^9^ spores/mL. A 1 mL aliquot of the spore suspension was inoculated into 50 mL of seed medium in a 250 mL flask and incubated at 30°C and 200 rpm for 24 h to produce the seed culture. Subsequently, 10% (v/v) of the seed culture was inoculated into 50 mL of fermentation medium in a 250 mL flask, and the fermentation was carried out at 30°C and 200 rpm for 48 h.

All media components were purchased from Sinopharm Chemical Reagent Co., Ltd. (Shanghai, China) at analytical and biochemical grades. All the media were sterilized in an autoclave at 121°C for 20 min.

### 2.2 Compound mutagenesis

**FIGURE 2:**
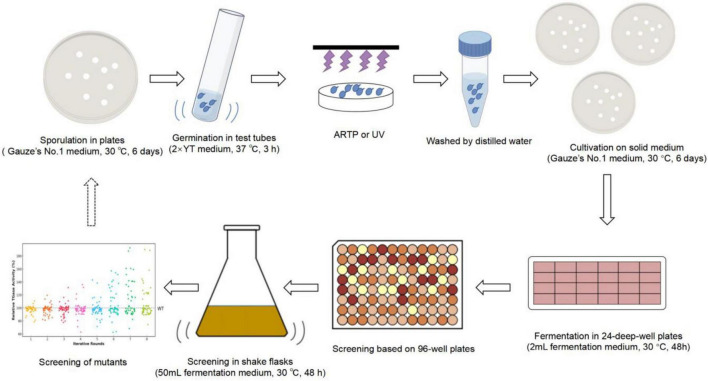
Scheme of ARTP–UV mutagenesis for microbial breeding.

The iterative mutagenesis and screening of the *S. mobaraensis* genome using the ARTP mutagenesis system (ARTP-IIS, Wuxi TMAXTREE Biotechnology Co., Ltd., Wuxi, Jiangsu, China) are shown in Figure 2. The method of operation was slightly modified from Han et al. (2022) and Yin et al. (2021). Spores were grown on Gauze’s No. 1 solid agar plates for 6 days and then transferred to 2 × YT for germination at 37°C for 3 h. The germinated spores were harvested and washed three times with sterile water. The lethality of ARTP and UV mutations was determined, and the time at which the lethality was 90% was selected as the time of mutation to ensure mutation efficiency. The treated spores were resuspended in an appropriate amount of sterile water and spread onto Gauze’s No. 1 solid agar plates. Single colonies were randomly selected from the solid medium and inoculated into 24-deep-well plates for fermentation, with a 48-h incubation period. The top 10 mutants with the highest TGase activity were selected for shake flask fermentation, and the mutant with the highest increase in TGase production was selected as the parent strain for the next round of mutagenesis. Eight rounds of mutagenesis were performed. The genetic stability of the identified mutants was evaluated by 8 generations of passaging.

### 2.3 SDS-PAGE electrophoresis

SDS-PAGE gels were prepared using a rapid SDS-PAGE gel preparation kit (Shandong Sparkjade Biotechnology Co., Ltd.), with a 10% concentration for the stacking gel. The electrophoresis procedure was carried out according to the manufacturer’s instructions. Following elecjnkmtrophoresis, the gel was stained with Coomassie Brilliant Blue R-250 until the bands appeared blue-black. The gel was then destained using a destaining solution until the background became nearly colorless. Finally, the gel was observed using a gel imaging system.

### 2.4 Scanning electron microscopy

For SEM analysis, fungal mycelia cultivated on Gauze’s No. 1 medium Petri dishes for 6 days were washed with potassium phosphate buffer (0.1 M, pH 7.2) and fixed in 5% glutaraldehyde. After fixation, the samples were stained with 1% osmium tetroxide. Subsequently, the fungal mycelia were dehydrated through a graded ethanol series of 0, 50, 70, 90, and 100% (v/v). Finally, the samples were coated with a platinum-palladium sputter coating and examined under a scanning electron microscope.

### 2.5 Establishment of the shake flask co-culture system

The seed culture used for the co-culture system must be fully activated. The SmGL spore suspension was prepared (about 10^8^ spores/mL), inoculated into the seed medium, and then incubated for 24 h to obtain the SmGL seed culture. Meanwhile, *B. amyloliquefaciens* CICC10888 was grown overnight, inoculated into 5% (v/v) LB medium, and grown until OD600 = 0.6. After reaching the desired optical density, the sample was diluted with sterile physiological saline (0.85% NaCl) to prepare the seed culture.

For experimental convenience, the inoculation ratio of both bacteria in the co-culture system in this study was set at 10% (v/v). In addition, the seed solution of *B. amyloliquefaciens* CICC10888 was diluted 2, 5, 10, 100, and 1,000 times before being inoculated into the co-culture system and then incubated in a shaking bed at 30°C and 200 rpm for 48 h.

### 2.6 Fermentation in a 2 L stirred-tank bioreactor

Fermentation was carried out in a 2 L glass stirred-tank bioreactor (T&J-MiniBox, T&J Bio-engineering Co., Ltd, Shanghai, China) with a working volume of 1.4 L. The specific parameters of the fermentation tank are listed in Supplementary Table S3. Prior to inoculation, the sterilized bioreactor was maintained at 30°C, with an aeration rate of 0.5 vvm and an agitation speed of 200 rpm. The initial pH was adjusted to 7 by manually adding ammonia water (12.5%, w/v). Once the system stabilized, the seed culture was inoculated into the bioreactor. Throughout fermentation, pH and dissolved oxygen (DO) were monitored online using pH and DO electrodes. The stirring speed was automatically adjusted to maintain DO at no less than 30%. When the stirring speed reached 800 rpm, the aeration rate was manually increased stepwise by 0.5 vvm, ranging from 0.5 to 2.5 vvm, to maintain DO above 30% until the end of fermentation. Samples were taken at regular intervals, and necessary measurements were performed during the process.

### 2.7 Methods for determination of TGase activity, dry cell weight, and glycerol content

All samples were centrifuged at 4500 × g for 10 min at 20°C. To determine the dry cell weight, the precipitates were collected and washed two times with deionized water, and then the washed mycelium was filtered through a pre-weighed filter paper and dried at 105°C to a constant weight. The supernatant was used for further analysis. The glycerol content was determined using a colorimetric method. TGase activity assay (Liu et al., 2011): reaction solution A consisted of 0.2 mol/L Tris-HCI (pH 6.0), 0.1 mol/L hydroxylamine, 0.1 mol/L glutathione, and 0.03 mol/L N-CBZ-Gln-Gly, and termination solution B consisted of 3 mol/L hydrochloric acid, 12% trichloroacetic acid, and 5% ferric chloride hexahydrate (dissolved in 0.1 mol/L hydrochloric acid) mixed in equal volume. For sample determination, 200 μL of TGase solution was added with 1 mL of reagent A, reacted at 37°C for 10 min, and then added with 1 mL of reagent B to terminate the reaction. Afterward, the sample was centrifuged at 4°C for 10 min at 7,000 r/min, and then the color was measured at 525 nm. In the control group, 200 μL of sample was initially added with reagent termination solution B and reacted at 37°C for 10 min and then added with reagent A. Other operations were the same. One unit of TGase activity was defined as the amount of enzyme required to produce 1 μmol of monohydroxamic acid per 1 min of the reaction at 37°C in unit of U/mL.

### 2.8 Quantitative real-time PCR analysis

qRT-PCR was used to analyze gene expression. Total RNA was extracted from the wild-type SmDL and mutant SmGL strains after 24 h of culture. RNA quality and quantity were assessed using a NanoDrop 2000c UV-visible spectrophotometer (Thermo Scientific, United States) and agarose gel electrophoresis. One gram microgram of RNA was treated with DNase I (Takara, China) to remove DNA contamination. cDNA was synthesized using the PrimeScript II 1st Strand cDNA Synthesis Kit (Takara, China) following the manufacturer’s instructions. The primers used are listed in Supplementary Table S1. qRT-PCR was performed using SYBR^®^ Premix Ex Taq™ II (TaKaRa, China) on a Line Gene 9600 Real-Time PCR system (Bioer, China) under the following conditions: 95°C for 30 s, then 40 cycles of 95°C for 5 s and 60°C for 20 s. Each sample was run in triplicate. A melting curve analysis was performed at the end of each run to verify specificity. Gene expression levels were normalized to the 16S rRNA gene, which was used as an internal control. The relative expression levels were calculated using the 2^−ΔΔCt^ method (Livak and Schmittgen, 2001). The melting curves are provided in Supplementary Figure S1.

### 2.9 Experimental procedures for genome sequencing

Strain sampling conditions: 30°C, 200 rpm culture for 48 h.

Sample quality inspection → Illumina TruSeq Nano DNA LT library construction (DNA fragmentation → DNA double-terminal repair → The introduction of an “A” base at the 3 ‘end → Joint connection → Purification of connected products → Library verification → Homogenization and multiplexing of libraries → Computer sequencing) → Evaluate original sequencing data by FastQC → Fragment DNA(150 bp) → Clean llumine Reads → Assembled Genome.

#### 2.9.1 Genome function annotation

The gene annotation software NCBI Blast+ was used to compare gene protein sequences with CDD, KOG, COG and other databases to obtain functional annotation information. GO function annotation information was obtained according to the annotation results of the gene, Swissprot and TrEMBL.

#### 2.9.2 Comparative genomic analysis

MUMmer (Kurtz et al., 2004) comparison software was used to detect and compare the single-nucleotide SNP (single-nucleotide variation) of the assembled individuals in the genome. MUMmer software was used to detect InDel mutations. SyRI (Goel et al., 2019) was used to detect the SV (structural variation).

### 2.10 Experimental procedures for transcriptome sequencing

Total RNA extraction → Detect the quality of total RNA → rRNA removal → RNA fragmentation → Synthesizing the first strand of cDNA → Synthesizing the second strand of cDNA → Detect the library size → Determine the total concentration of the library → Using illumina platform to complete the sequencing.

#### 2.10.1 Analysis of RNA-seq data and differential expression genes analysis

The pretreated original data were subjected to bowtie2 (Langmead et al., 2009) (version: 2-2.0.5) for genome comparison. HTSeq (Anders et al., 2014) was used to count the number of fragments of each gene after bowtie2 comparison, and TMM (Robinson and Oshlack, 2010) was used to normalize the data. Finally, perl script was used to calculate the FPKM (Mortazavi et al., 2008) values of each gene. edgeR (Robinson et al., 2010) was applied to analyze the differences among samples. The p-value threshold was determined by controlling the FDR (Yoav and Daniel, 2001), and the differential expression multiple (fold-change) was calculated according to the FPKM value. The screening conditions of differential genes were a q-value (corrected p-value) of ≤ 0.05 and fold-change of ≥ 2, and the analysis of differentially expressed genes mainly consisted of GO enrichment analysis.

#### 2.10.2 Functional enrichment analysis of differentially expressed genes

Top GO was used for GO enrichment analysis. During the analysis, the differential genes annotated by GO terms were used to calculate the gene list and gene number of each term. Then, the hypergeometric distribution method was used to calculate the p-value (the standard for significant enrichment was a *p*-value of < 0.05) and compare with the whole genome background. According to the GO enrichment analysis results of differentially expressed genes, GO classification was carried out according to biological process BP, molecular function MF, and cell component CC, and the GO function classification annotations of differentially expressed genes were given. The top 10 GO term entries with the lowest *p*-value, that is, the most significant enrichment, were selected from each GO classification for display.

The genome sequencing mentioned above was conducted by Shanghai Peisenol Biotechnology Co., LTD., while the transcriptome sequencing was performed by Sangon Biotech (Shanghai) Co., LTD., the main software and databases are listed in Supplementary Table S2, using three replicates per analysis.

### 2.11 Statistical analyses

Each value is presented as the mean of three replicates, expressed as mean ± standard deviation (mean ± S.D.). Statistical analyses were performed using GraphPad Prism 9.0. Differences between the experimental and control groups were assessed using Student’s *t*-test. For all comparisons, a *p*-value of < 0.05 was considered statistically significant.

## 3 Results and analyses

### 3.1 UV–ARTP combined mutagenesis for strain breeding

Bacterial mortality increased with the increase of ARTP and UV treatment time. When the wild strain SmDL was treated with ARTP for 50 s and UV light for 60 s, the lethality rates were 90.91 and 92.89%, respectively (Supplementary Figures S2, S3). According to previous reports, treatment time that results in a lethality rate of approximately 90% is typically used to ensure the efficiency of mutagenesis (Guo et al., 2011). Therefore, 50 s of ARTP treatment and 60 s of UV treatment were applied in this study.

**FIGURE 3:**
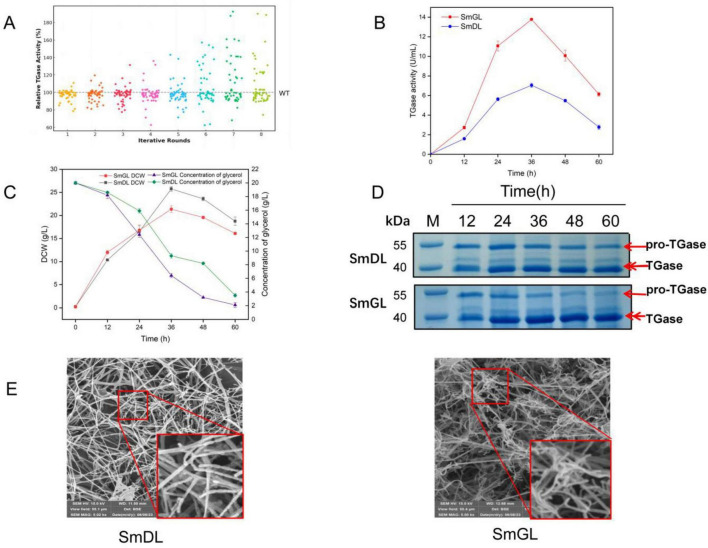
Performance and morphology of high-yielding mutant strains. **(A)** Distribution of results from each round of mutation screening. **(B)** Time course of TGase production. **(C)** Time course of bacterial dry weight and glycerol content. **(D)** SDS-PAGE analysis of culture supernatants at different time points during fermentation. Channel M, protein marker. Samples were taken from 12, 24, 36, 48, and 60 h. The full gel image, showing the entire molecular weight range, is provided in Supplementary Figure S5. **(E)** Scanning electron microscopy of mycelial differentiation on a solid medium. smDL and smGL were cultured on Gauze’s synthetic medium No. 1 at 30°C for 6 days. Spores were shown in squares.

A total of 400 mutants were screened through eight rounds of mutagenesis, followed by shake flask fermentation for verification. Finally, a high-yielding strain, namely, SmGL, was selected from the 400 mutant strains. As shown in Figure 3A, the TGase activity of the high-yielding strain SmGL was increased by 92.43% compared with that of the wild strain SmDL, with a TGase activity of 7.12 U/mL. The TGase activity of the authenticated mutant SmGL remained stable after 8 rounds of passaging (Supplementary Figure S4).

### 3.2 Fermenter production testing of high-yield mutant strains

By comparing the TGase activity, glycerol content, and dry weight of the bacteria in the 2 L fermenter (Figures 3B,C), the TGase activity of SmGL was increased by 95.31% to 13.77 U/mL by multiple rounds of mutagenesis compared with that of SmDL in the fermenter, and the mutant bacteria consumed the glycerol content more rapidly during fermentation. In addition, the glycerol residue was lower than that of the control group, and the biomass was slightly higher than that of the control group in the first 24 h but lower than the control group after 24 h. We hypothesized that mutagenesis has changed the metabolic pathway and the direction of metabolic flow of the strain to a certain extent, and that more substances and energy are supplied to the biosynthetic pathway of TGase more rapidly than that of the original strain. Protein electrophoresis analysis revealed that the accumulation of pro-TGase in SmGL is visibly lower than that in SmDL, but a distinct pro-TGase band is still observed at the end of fermentation (Figure 3D). This finding suggests that the activation of TGase is not yet complete at the end of fermentation.

### 3.3 Morphological changes of high-yielding mutants

Based on SEM observations, compound mutagenesis also induced morphological changes in the strain (Figure 3E), where SmGL grew on a solid medium with more branched and curled spore chains and significantly increased the number of spores compared with the wild strain SmDL. Previous studies on the correlation between *S. mobaraensis* mycelial morphology and TGase synthesis are limited, whereas several studies have been conducted on the correlation between the morphology and product synthesis in other Streptomyces. Numerous studies have shown that the synthesis of metabolites such as Streptomyces proteins is positively influenced by spore formation, such as pB, Chaplins, and Rodins proteins (Flardh and Buttner, 2009). In a later study, considerable evidence has shown that the morphological changes in *S. mobaraensis* affect TGase synthesis [see section 3.5.1, (4)]. In this study, the observed morphological alterations had a significant impact on TGase production in *S. mobaraensis*, further supporting the role of morphological differentiation in enhancing enzyme yields.

### 3.4 Genomic analysis

**FIGURE 4:**
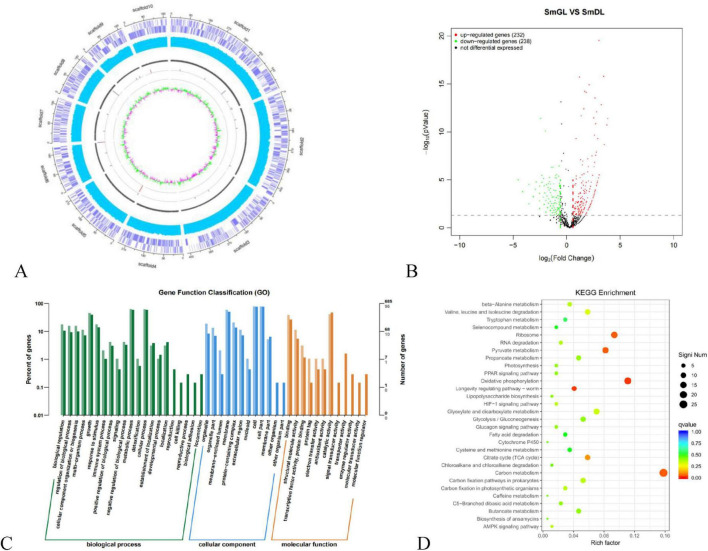
Comparative genomic and transcriptomic data. **(A)** Genomic variation mapping. **(B)** Volcano mapping of significantly and differentially expressed genes in SmGL and SmDL. Red indicates upregulated genes; green indicates downregulated genes, and black indicates non-differentially expressed genes. **(C)** GO functional annotation. **(D)** Statistical features of DEG enrichment in the KEGG pathway.

To reveal the relationship between phenotypic and genetic variation and to further investigate the molecular mechanisms of the TGase synthesis pathway, we used SmDL and SmGL for comparative genomic analysis. Genome-wide comparisons were performed between the high-yielding TGase mutant SmGL and the original SmDL strain, and genomic variation mapping revealed microstructural mutations between SmGL and SmDL (Figure 4A). The increase in TGase production induced by compound mutagenesis can be attributed to two effects: a direct effect if the structural gene of the TGase zymogen (pro-TGase) is altered, resulting in a change in the specific activity of the protein, and an indirect effect when mutations occur in sequences related to the regulation of TGase zymogen gene expression. Compared with the SmDL genome, 95 mutations were identified in the SmGL genome, including 92 single-base mutations, two insertion mutations, and one deletion mutation. The sequences of the mutated genes were compared with the reference genome sequences in the KEGG functional annotation database. Although the functions of some genes are unknown, some of the genes with base mutations are functionally defined (Table 1), and they can be categorized in accordance with their functions in amino acid metabolism (*bkdA*, *argJ*, and *argC*), lipid metabolism (*mbtM*), nucleotide metabolism (*tmk*), and genetic information processing (*k07482*). The sequences of TGase zymogen (pro-TGase) and TGase activation-associated encoded proteins [TAMEP, tripeptidyl aminopeptidase (TAP), and P14] were identical before and after the mutation. Moreover, no change was observed within the 2000-base region upstream of the abovementioned genes. Therefore, the expression of the abovementioned genes and the encoding process were not the direct cause of the changes in TGase fermentation.

**TABLE 1:** KEGG analysis and type of genetic mutation.

Gene name	Mutation type	KEGG functional description
*bkdA*	SNP	2-oxoisovalerate dehydrogenase E1 component alpha subunit
*tmk*	SNP	dTMP kinase
*mbtM*	SNP	Long-chain-fatty-acid–[acyl-carrier-protein] ligase
*argJ*	SNP	Glutamate N-acetyltransferase/amino-acid N-acetyltransferase
*K07482*	SNP	Transposase, IS30 family
*argC*	SNP	N-acetyl-gamma-glutamyl-phosphate reductase

Base mutations can be classified into missense and nonsense mutations based on whether they result in a change in the amino acid sequence. The percentage of arginine in the TGase amino acid sequence is 8.85%, which is higher than the average. Thus, we focused on the missense mutation with number gene 6432, which occurs in ArgJ (arginine biosynthesis bifunctional protein). ArgJ is an enzyme involved in the arginine biosynthesis pathway, specifically in the conversion of glutamate to arginine. As a bifunctional enzyme, ArgJ catalyzes key steps in this process, playing an essential role in nitrogen metabolism. Its activity is crucial for maintaining cellular functions, such as protein synthesis and nitric oxide production, by regulating arginine levels within the cell (Wang et al., 2022). Meanwhile, two missense mutations occurred in the coding sequence, leading to changes in the corresponding amino acids. Using the Alphafold 3 protein modeling tool, these mutation sites are spatially distant from the catalytic center and key structural regions (Supplementary Figure S6). Therefore, directly concluding that these mutations impact arginine or TGase synthesis is difficult.

Synonymous mutations are not without effect. The redundancy of the genetic code indicates that most amino acids are encoded by multiple synonymous codons. Although translation initiation is crucial, codon bias improves efficiency by affecting elongation. Preferred codons speed up translation, while less common codons slow it down, influencing overall protein synthesis and folding (Quax et al., 2015); thus, codon bias plays an important role in controlling various cellular processes, from differential protein production to protein folding (Kudla et al., 2009). Combined with the data from the Codon Frequency Analysis Database,^1^ 30 codon mutations, accounting for 54.54%, were used at high frequency by synonymous mutations to *S. mobaraensis* in this study. Notably, some genes have mutations toward codons that are frequently used by *S. mobaraensis*, for example, 16 synonymous mutations in gene5088 and 11 mutations toward high-frequency codons, and three mutations in gene6040, all of which are toward high-frequency codons. On the contrary, some genes have mutations toward codons that are less frequently used, for example, four out of five mutations in gene2057 are toward low-frequency codons, and five out of six mutations in gene 3640 are toward low-frequency codons. Synonymous codon mutations can directly or indirectly affect the synthesis of related products, as they have a deeper impact on translation and protein folding efficiency, particularly in the high-level expression of secreted or membrane proteins (Angov et al., 2008; Oelschlaeger, 2024). Over the past four decades, extensive research has been conducted on codon usage bias, with several insights widely applied in biotechnology as strategies to optimize gene expression and enhance protein productivity and yield. This study offers an excellent approach to refining the “codon usage bias” method to improve TGase activity and to design and express TGase heterologs.

### 3.5 Transcriptomic analysis

Based on the TGase activity and growth curves of the experimental groups SmGL and control SmDL, 24 h in the middle of the logarithmic phase of fermentation was selected as the sampling time of transcriptome samples. Based on the RNA-Seq sequencing data, differential gene expression analysis was performed using DESeq2 to screen differential genes and analyze the differences in gene expression levels, and the results of differential expression analysis were visualized.

The volcano plot reveals significant differences in gene and transcript expression levels between two sample groups. This visualization allows for macroscopic observation of fold changes, highlighting upregulated and downregulated genes (Figure 4B). Following 24 h of fermentation, the mutant strain SmGL exhibited 232 upregulated genes and 238 downregulated genes compared with the original strain SmDL.

These transcriptomes were classified using Gene Ontology (GO), Kyoto Encyclopedia of Genes and Genomes, and KEGG pathway analyses. GO analyses revealed significant enrichment of terms in various biological processes (Figure 4C), whereas KEGG analyses revealed that ribosomes, oxidative phosphorylation, pyruvate metabolism, carbon metabolism, and TCA cycle (Figure 4D) were enhanced. These results indicate that 95 base changes by compound mutagenesis directly or indirectly led to the entire metabolic network related to cellular metabolic processes, biosynthetic processes, and energy metabolism in *S. mobaraensis*.

### 3.6 Analysis of major differential genes in the transcriptome

In obtaining insights into the causes of increased TGase activity, differentially expressed genes were analyzed in depth. The major differential genes are listed in Supplementary Table S4. Although no significant differences were found in the expression of the TGase synthesis genes, some related expressions may be responsible for the increased TGase activity.

#### 3.6.1 Differentially expressed genes involved in carbon metabolism

**FIGURE 5:**
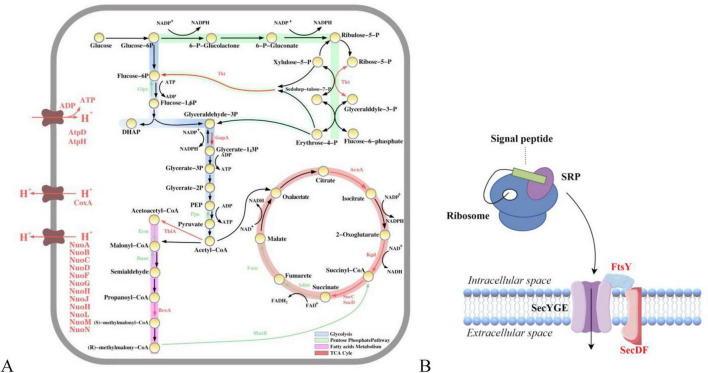
Map of metabolic pathway transcriptional level differences and transmembrane transport mechanisms. **(A)** Annotation of key differential proteins involved in central carbon metabolism. Red arrows (upregulated), green arrows (downregulated), and black arrows (no significant change). **(B)** Sec pathway. Black (no significant change) and red (upregulated).

Glycolysis, the pentose phosphate pathway, fatty acid metabolism, and the TCA cycle are the four pillars of cellular metabolism, and these pathways play crucial roles in energy production, carbon source utilization, and biosynthesis. Glycolysis provides direct energy to the cell by catabolizing glucose to produce pyruvate and generating ATP and NADH. In addition, the pentose phosphate pathway is a key pathway for the production of nucleotides and amino acids, and it provides the cell with reducing power (Stincone et al., 2015). Fatty acid metabolism generates large amounts of ATP through β-oxidation, and the TCA cycle is an important part of cellular respiration, providing an electron donor for oxidative phosphorylation through the oxidation of pyruvate and other metabolic intermediates to produce NADH and FADH_2_ (Fernie et al., 2004). Four metabolic pathways, namely, glycolysis, pentose phosphate, fatty acid metabolism, and the TCA cycle, were upregulated with essential enzyme genes, and sugar utilization was enhanced in the mutant strains, indicating that mutagenesis collectively enhanced the four abovementioned pathways (Figure 5A). In addition, cellular respiration promotes the formation of ATP through oxidative phosphorylation, which serves as the main energy carrier in cellular processes (Kaila and Wikström, 2021). Moreover, the increased synthesis of respiratory chain enzymes (e.g., NADH dehydrogenase NuoABCDFGHJHLMN, cytochrome reductase ISP, cytochrome oxidase CoxA, and ATP synthetase AtpAH) could promote bacterial growth and TGase production (Zhao et al., 2014). These findings indicate that energy transfer and carbon source utilization during microbial metabolism are altered in the highly productive strain SmGL, which plays a key role in TGase synthesis. Although the transcriptional changes do not directly indicate TGase synthesis, they form the energy basis for the synthesis, activation, and transport of TGase in the mutant strain.

#### 3.6.2 Differentially expressed genes involved in protein export

The TGase produced by *S. mobaraensis* is an excretory protein. TGase exists in the form of a pre-pro-TGase, which is a precursor protein with a signal peptide, inside the cell. It undergoes transmembrane transport, where the signal peptide is recognized and cleaved, and then it is transported to the extracellular space as the pro-TGase precursor protein (Kanaji et al., 1993). The secretion pathway of *S. mobaraensis* is not fully understood. In this study, genes related to the high-yield TGase secretion signal recognition particle (Sec-SPR) pathway were found to be significantly upregulated. Therefore, this protein transport system is a crucial pathway for the transport of TGase (Figure 5B; Pohlschröder et al., 2005; Driessen and Nouwen, 2008). In general, SecD and SecF form the SecDF complex, which stabilizes and enhances the transmembrane transport of proteins by utilizing the proton motive force to assist in the release of proteins from the SecYEG channel to the other side of the membrane (Cascales et al., 2017). The signal recognition particle (SRP) binds to the signal peptide on the nascent polypeptide chain and interacts with the SRP receptor FtsY, directing the complex to the SecYEG transporter channel in the cell membrane, thereby facilitating the co-translational translocation of proteins (Pyrih et al., 2021). The upregulation of the transcriptional levels of SecD and FtsY proteins also results in the upregulation of ABC transporter protein-related expression, an interaction that increases membrane permeability and triggers phosphorylation, thereby enhancing the signaling process (Liang et al., 2020). This phenomenon not only improves the efficiency of intracellular-to-extracellular transport but also reduces potential cytotoxicity, thereby decreasing intracellular protein aggregation (Holland and Blight, 1999). This interaction is achieved through intercellular regulation of these proteins, which directly or indirectly play a role in pro-TGase transport and regulation, whereas the upregulation of transcript levels of enzymes related to carbon metabolic pathways ensures an adequate energy supply.

#### 3.6.3 Differentially expressed genes involved in pro-TGase somatically activated protease

S. *mobaraensis* initially secretes pro-TGase in its zymogen form. The zymogen region, which is located at the N-terminus of the active enzyme and consists of 45 amino acids, is crucial for inhibiting enzymatic activity while facilitating the efficient folding and secretion of the enzyme (Yurimoto et al., 2004). During the simultaneous secretion of two proteases involved in zymogen activation, including TAMEP and TAP, TAMEP initially excises the first 41 amino acids of the zymogen region, generating FRAP TGase, and then TAP excises FRAP to generate TGase (Figure 1C; Zotzel et al., 2003a; Zotzel et al., 2003b). Based on the transcriptome results, the transcriptional level of TAP was significantly upregulated in the mutant SmGL, which was consistent with the protein electrophoresis results that showed a significant enhancement of the TGase bands in the mutant between 24 and 60 h and a concomitant weakening of the pro-TGase bands (Figure 3C), indicating that TAP plays a critical and positive role in TGase activation. According to Yin et al. (Yin et al., 2022), the addition of NH_4_^+^ can enhance the protease activity in *S. mobaraensis* compared with no NH_4_^+^, which resulted in a 2.1-fold increase in TGase productivity and a 43% reduction in maximum yield. However, Zhang (2012) study, the addition of EDTA (TAMEP inhibitor) and PMSF (TAP inhibitor) to the medium showed that the main protease involved in the activation of pro-TGase was TAMEP, whereas TAP played a relatively minor role, which is inconsistent with the results of this paper, that is, the upregulation of the transcriptional level of TAP protease led to the increase in the enzymatic activity of TGase. Both this study and previous research indicate that the activation of pro-TGase is a key factor in enhancing TGase activity, although there are some differences in the studies regarding the activation of the main proteases involved in the specific activation mechanisms. We also failed to identify potential mutations that could upregulate the TAP transcript levels in comparative genomic analyses, suggesting a large number of questions surrounding the activation of TGase and the regulation of its associated genes that remain to be investigated.

#### 3.6.4 Differentially expressed genes involved in spore formation

The significant 21.18-fold upregulation of SigmaE transcription, along with the increased transcription of Spore-associated protein A, highlights a critical link between stress response and sporulation in *S. mobaraensis*. As noted, this upregulation supports cell growth under stressful conditions, contributing to the enhanced sporulation capacity of the mutant strain SmGL (Figure 1E). Previous studies have demonstrated that sporulation in actinomycetes is not merely a self-replicative process but also a response to environmental factors, impacting cellular metabolism within the spore envelope (Yagüe et al., 2013). This aligns with our findings, where SigmaE plays a vital role in synthesizing the spore cortex and protective layers, facilitating spore formation even under nutrient-limited conditions (Busche et al., 2022).Moreover, SigmaE’s role extends beyond sporulation; it also regulates membrane stress responses, integrating signals from thermal, oxidative, and DNA damage-related stresses, and protecting bacterial cells from environmental threats (Ehira et al., 2009). Given that spore formation has been linked to metabolic activity, the upregulation of SigmaE may also have implications for TGase synthesis. The regulatory network controlled by SigmaE could enhance TGase production by ensuring cell survival and function in adverse conditions, making this pathway a promising target for future studies focused on optimizing TGase yield in industrial applications. In summary, the findings of this study suggest that the increased sporulation and the associated SigmaE-mediated stress response may play a crucial role in enhancing TGase synthesis, providing a basis for further exploration of the interplay between spore formation and enzyme production.

### 3.7 Genome-combined transcriptomic analysis

Mutations in the bases encoding the genome are often accompanied with changes in the level of transcription of the gene, which affect the protein encoded by the gene, thereby influencing cell growth, cellular functions, and product formation. The methylerythritol phosphate (MEP) pathway produces terpene precursor molecules, and IspG is the rate-limiting enzyme of the MEP pathway, with various cellular functions ranging from basic roles such as maintaining membrane fluidity and supporting electron transport to highly specialized roles such as hormone signaling and cellular defense (Sell, 2000). Mutations in the gene coding for IspG have also downregulated the transcriptional level of the gene, which may cause membrane permeability changes and promote TGase efflux. Notably, in the amino acid metabolic pathway, the two coding genes of arginine metabolism (*argC* and *argJ*) were mutated, and the transcription level of the *argJ* gene was downregulated. Translating too quickly or too slowly can affect the folding and coiling of the polypeptide chain. For example, the abovementioned arginine accounted for a large proportion of the amino acid sequence of TGase, and the downregulation of the transcription level of genes related to the arginine synthesis pathway might affect the translation speed of TGase, further affecting TGase activity.

Our integrated genomic and transcriptomic analyses suggest a hypothetical mechanism in which specific mutations contribute to increased TGase activity in the high-yield mutant *S. mobaraensis* SmGL. Genomic mapping revealed mutations, notably in pathways related to amino acid metabolism (e.g., *argC*, *argJ*) and cellular metabolism (*ispG*), which, when combined with transcriptomic data, indicate potential regulatory effects on TGase production. The transcriptional upregulation of genes associated with energy and carbon metabolism pathways (e.g., glycolysis, TCA cycle) likely provides a robust metabolic and energetic basis for enhanced TGase synthesis and secretion. Furthermore, upregulated expression in protein transport and processing pathways, such as the Sec-SPR pathway, may facilitate efficient TGase export, while protease-related transcripts indicate increased TGase activation efficiency.

This evidence supports a mechanistic hypothesis that specific mutations drive metabolic and regulatory adaptations, collectively enhancing TGase synthesis, activation, and secretion in the mutant strain. Based on the above perspective, we believe that TG-related genes are non-essential for the strain’s survival. Therefore, the expression levels of these genes may have greater uncertainty, which is precisely the basis on which our study was initiated.

### 3.8 Validation of qRT-PCR

In verifying the accuracy of the transcriptome sequencing data, samples from 24 h of fermentation were collected for qRT-PCR validation, and six genes were screened (Supplementary Figure S7). Despite some discrepancies, qRT-PCR data were basically the same as the transcriptome data, indicating that the transcriptional sequencing data were reliable.

### 3.9 Improving the production of TGase by co-culture with SmGL and B. amyloliquefaciens CICC10888

Based on the protein gel electrophoresis images, the pro-TGase to TGase band and the upregulation of TAP transcription levels indicate that the activation of pro-TGase is crucial for increasing TGase activity. Although the upregulation of TAP transcription levels may have contributed to the increased TGase activity, Figure 3D still shows that some pro-TGase remains unconverted to TGase, indicating the further enhancement of TGase activity. Considering the public opinion risks associated with gene editing, “non-genetic” strategies are more readily accepted by the food industry (Yunes et al., 2021). In this study, proteases (neutral protease, trypsin, and pancreatic rennet protease) were found to activate pro-TGase by reviewing the literature (Yang et al., 2009; Supplementary Figure S8). After comparing the activation efficiency of several proteases, neutral protease was the most effective (Supplementary Figure S9), and the excellent neutral protease-producing strain *B. amyloliquefaciens* CICC10888 was selected for co-culture, the *B. amyloliquefaciens* CICC10888 strain is a widely used industrial strain in China. It was developed last century through traditional mutagenesis methods, as well as optimization of fermentation parameters and culture media and is characterized by its high capacity for neutral protease production (Wang et al., 2016). In the optimization experiment (Supplementary material S3.9), the most effective methods were determined for liquid seed inoculation of two bacterial strains.

**FIGURE 6:**
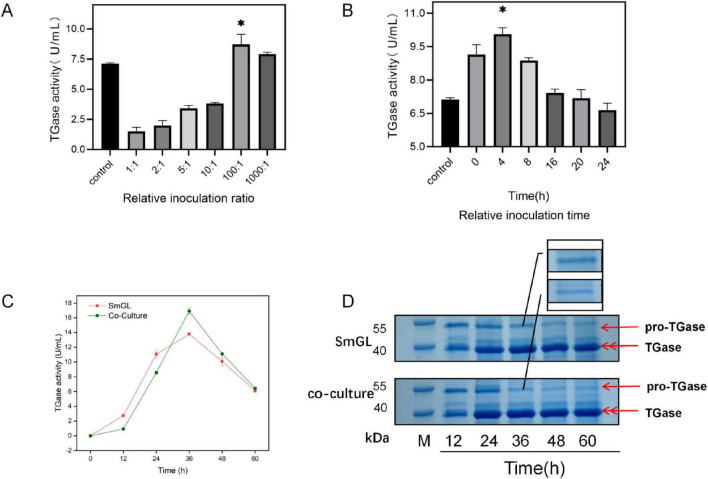
Optimization of co-culture fermentation conditions for increasing TGase activity in flask culture levels. **(A)** Relative inoculation rate. **(B)** Relative inoculation time (*P < 0.05). **(C)** Time course of TGase production in co-culture. **(D)** SDS-PAGE analysis of culture supernatants at different time points during fermentation in co-culture. The upper gel shows SmGL, and the lower gel shows the co-culture. Channel M represents the protein marker. Samples were taken at 12, 24, 36, 48, and 60 h. The full gel image, showing the entire molecular weight range, is provided in Supplementary Figure S5.

Figure 6C summarizes the TGase activity during fermentation in a 2 L fermenter. We found that TGase activity was lower than that of pure culture from 0 to 24 h, but between 12 and 36 h, TGase rapidly accumulated. In the early stage, some of the nutrients were directed toward *B. amyloliquefaciens* CICC10888. SDS-PAGE showed that the pro-TGase band became weaker at 36 h (Figure 6D). This conclusion strongly corroborates our omics analysis data and its reasonable inferences. The bottleneck in TGase synthesis lies in the post-translational modification. The co-culture technique remarkably enhances TGase fermentation activity.

Given the growing applications and significant market potential of TGase (Kolotylo et al., 2023), researchers have adopted various strategies to enhance TGase activity. These include optimizing the composition of culture media (Kolotylo et al., 2024), improving stirring conditions in tank fermentation (Yan et al., 2005) and utilizing genetic engineering approaches (Ye et al., 2024). Notably, Yuan et al. (2024b) achieved the highest reported TGase activity of 65.34 U/mL in a 1,000 L fermentation tank. In the present study, TGase activity was increased to 16.9 U/mL through co-culture fermentation, which is still below the current mainstream levels. However, this study identified potential genes in mutant strains that may further enhance TGase activity and employed the novel co-culture technology to improve enzyme activity. These findings provide valuable guidance for future efforts to boost TGase activity. As single strategies for enhancing TGase activity often yield limited results, further improvements in enzyme activity are expected through continued optimization in future studies.

## 4 Conclusion

To explore the intricate regulatory mechanisms involved in TGase synthesis by *S. mobaraensis*, this study utilized UV-ARTP combined mutagenesis to obtain a high-yield strain with stable inheritance, achieving a maximum TGase activity of 13.77 U/mL, accounting for a 92.43% increase. This strain served as a positive mutant gene pool, and comparative genomic and transcriptomic techniques were used to identify potential key regulatory factors. Comprehensive analysis indicates that changes in codon preference, amino acid metabolism, carbon metabolism, protein export, TGase activation, and spore formation collectively contribute to the enhancement of TGase activity. Subsequently, co-culture technology was applied with *B. amyloliquefaciens* CICC10888, which secretes neutral proteases to enhance the in vitro activation efficiency of TGase. The TGase activity reached 16.91 U/mL, reflecting an increase of 22.71%. This study provides valuable insights and data references for future research on TGase synthesis.

## Data Availability

The datasets presented in this study can be found in online repositories. The names of the repository/repositories and accession number(s) can be found at: PRJNA1172573 (SRA).

## Footnotes

1
https://www.bioinformatics.org/sms2/codon_plot.html
