# Supplementary material for: Multi-omics investigation of high-transglutaminase production mechanisms in Streptomyces mobaraensis and co-culture-enhanced fermentation strategies
Source: Front Microbiol. 2025 Feb 5;16:1525673. doi: 10.3389/fmicb.2025.1525673 (PMC11835810; doi:10.3389/fmicb.2025.1525673)
Supplement: Supplementary file 1 [file Data_Sheet_1.DOCX]

Supplementary Material

# Supplementary Figures and Tables

## Supplementary Tables

**Table. S1** Specific primers of gene sequence for qRT-PCR

| **Primer name** | **Primer sequence** | **Fragment size** |
| --- | --- | --- |
| 16s rRNA-F | 5' GGGCAGGCTAGAGTTCGGTAGG 3' | 140bp |
| 16s rRNA-R | 5' GTTCGCTCCCCACGCTTTCG 3' |  |
| *tap*-F | 5' AAGCCCATCCAGTGCGGTTG 3' | 86bp |
| *tap*-R | 5' GACACGGTCCACGGCGATC 3' |  |
| *atpA*-F | 5' GAGGAGGTCGGTACGGTCAGC 3' | 137bp |
| *atpA*-R | 5' TTCAGCAGCTCGTTCGCCATG 3' |  |
| *nuoA*-F | 5' CAAGTACTACCTGACGGCGATGC 3' | 99bp |
| *nuoA*-R | 5' CCGAACAGCCCGAGGGAGTC 3' |  |
| *secD*-F | 5' TTCGGTATGGAGTCGCTGTT 3' | 116bp |
| *secD*-R | 5' CTCTTGTCGCCCTTGTCCTTGTC 3' |  |
| *ftsY*-F | 5' GGCACGAGAAGGTGATGGA 3' | 90bp |
| *ftsY*-R | 5' GTTGACGCCGACGACCATGAC 3' |  |
| *sigE*-F | 5' CGCCGCACCATGACCAACC 3' | 94bp |
| *sigE*-R | 5' GGTCGCCCGCCGTCTCC 3' |  |

**Table. S2** Main software and databases

| **Software/Database** | **Website** |
| --- | --- |
| Fastp | https://github.com/OpenGene/fastp |
| AdapterRemoval | https://github.com/MikkelSchubert/adapterremoval |
| SOAPec | https://help.rc.ufl.edu/doc/SOAPec |
| A5-MiSeq | https://arxiv.org/abs/1401.5130 |
| SPAdes | http://cab.spbu.ru/files/release3.12.0/manual.html |
| SOAPdenovo | https://github.com/aquaskyline/SOAPdenovo2 |
| Pilon | https://github.com/broadinstitute/pilon |
| GeneMarkS | http://topaz.gatech.edu/GeneMark/ |
| Barrnap | http://www.vicbioinformatics.com/software.barrnap.shtml |
| tRNAscan-SE | http://lowelab.ucsc.edu/tRNAscan-SE/ |
| CRISPRCasFinder | https://github.com/dcouvin/crisprcasfinder |
| diamond | http://github.com/bbuchfink/diamond |
| Blast | https://blast.ncbi.nlm.nih.gov/Blast.cgi |
| TMHMM | http://www.cbs.dtu.dk/services/TMHMM/ |
| Blast | https://blast.ncbi.nlm.nih.gov/Blast.cgi |
| diamond | http://github.com/bbuchfink/diamond |
| hmmscan | http://hmmer.org/ |
| BLAST2GO | https://www.blast2go.com/ |
| map2slim | https://metacpan.org/pod/distribution/go-perl/scripts/map2slim |
| KAAS | https://www.genome.jp/tools/kaas/ |
| Rfam | http://rfam.xfam.org/ |
| VFDB | http://www.mgc.ac.cn/VFs/main.htm |
| CARD | <https://card.mcmaster.ca/> |
| CAZy | <http://www.cazy.org/> |
| BLAST2GO | <https://www.blast2go.com/> |
| map2slim | <https://metacpan.org/pod/distribution/go-perl/scripts/map2slim> |
| KAAS | <https://www.genome.jp/tools/kaas/> |
| Rfam | <http://rfam.xfam.org/> |
| NR | <ftp://ftp.ncbi.nih.gov/blast/db/> |
| eggNOG（COG） | <http://eggnogdb.embl.de/#/app/home/> |
| KEGG | <http://www.genome.jp/kegg/> |
| Swiss-prot | <http://www.uniprot.org/> |
| GO | <http://www.geneontology.org/> |

**Table. S3** 2L Fermenter Specific Parameters

| **Parameter** | **Value** |
| --- | --- |
| Impeller Type | Rushton2 + Pitched1 + AF Impeller*1 |
| Distance between impellers (mm) | 46 (Factory default distance equals one impeller diameter) |
| Vessel inner diameter (mm) | 114 |
| Vessel height (mm) | 228 |
| Vessel diameter to impeller outer diameter ratio | 0.4 |
| Number of baffles | 4 |
| Baffle dimensions (mm) | 11*160 |

**Table. S4** Major differential genes

| Upregulated genes | Downregulated genes |
| --- | --- |
| *atpD atpH coxA nuoA nuoD nuoB nuoC nuoF nuoG nuoH nuoJ nuoL nuoM nuoN tkt gapA thlA bceA acnA kgd sucC sucD ftsY secD tap sigE ispG* | *glpx ecm banC pps func mutB sdhB* |

## Supplementary Figures


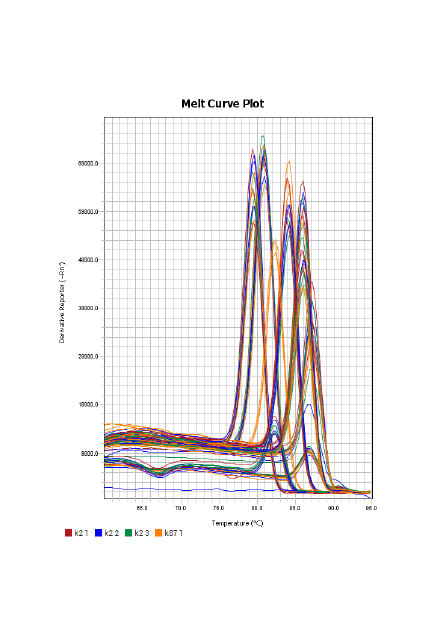


**Figure S1** Melting curve plot of qRT-PCR reactions for the analysis of gene expression.

**
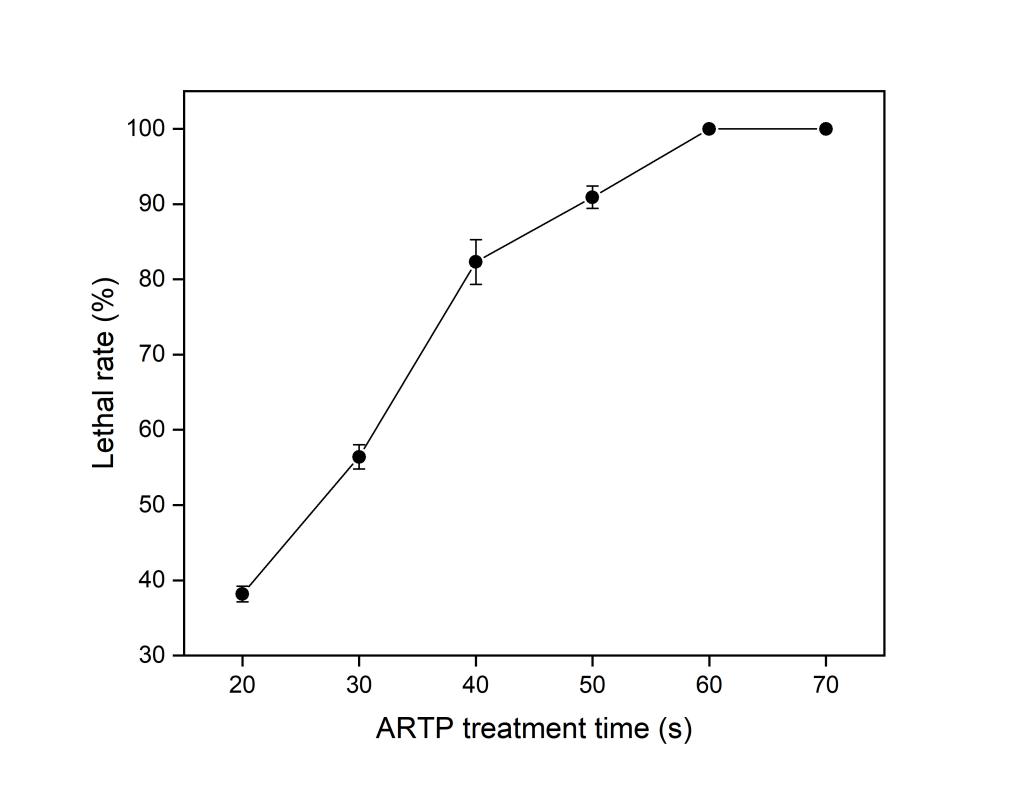
**

**Fig. S2** Lethal rate of S. mobaraensis by ARTP mutagenesis

**
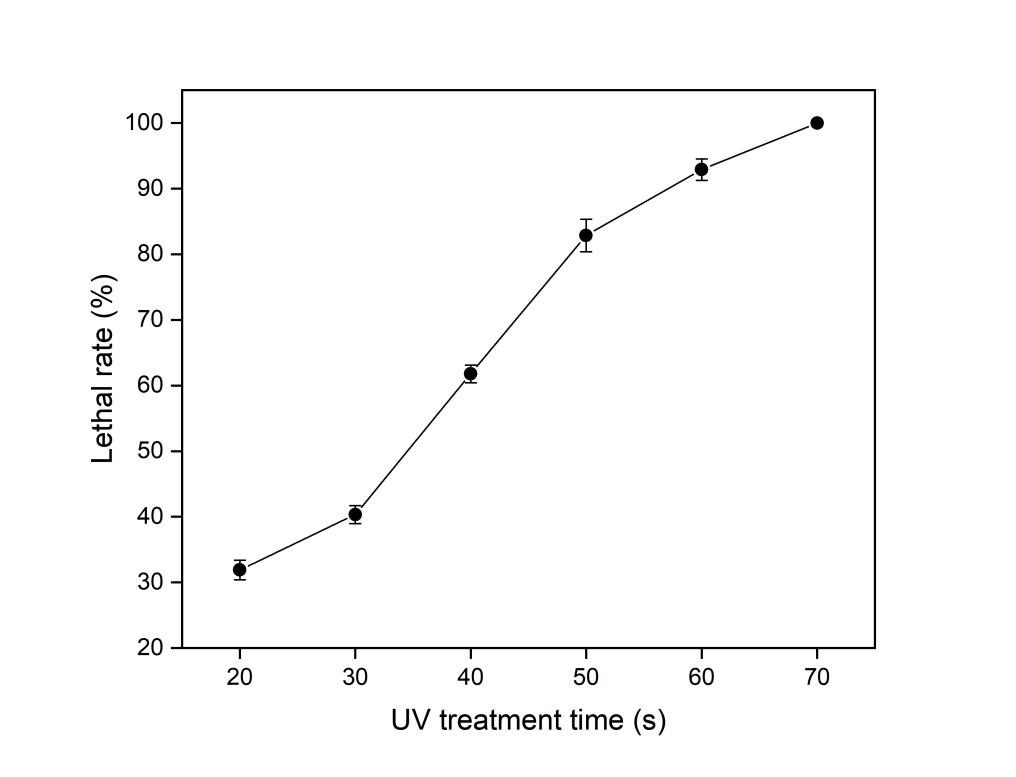
**

**Fig. S3** Lethal rate of S. mobaraensis by UV mutagenesis

**

**

**Fig. S4** Genetic stability verification in shake-flask fermentation

**
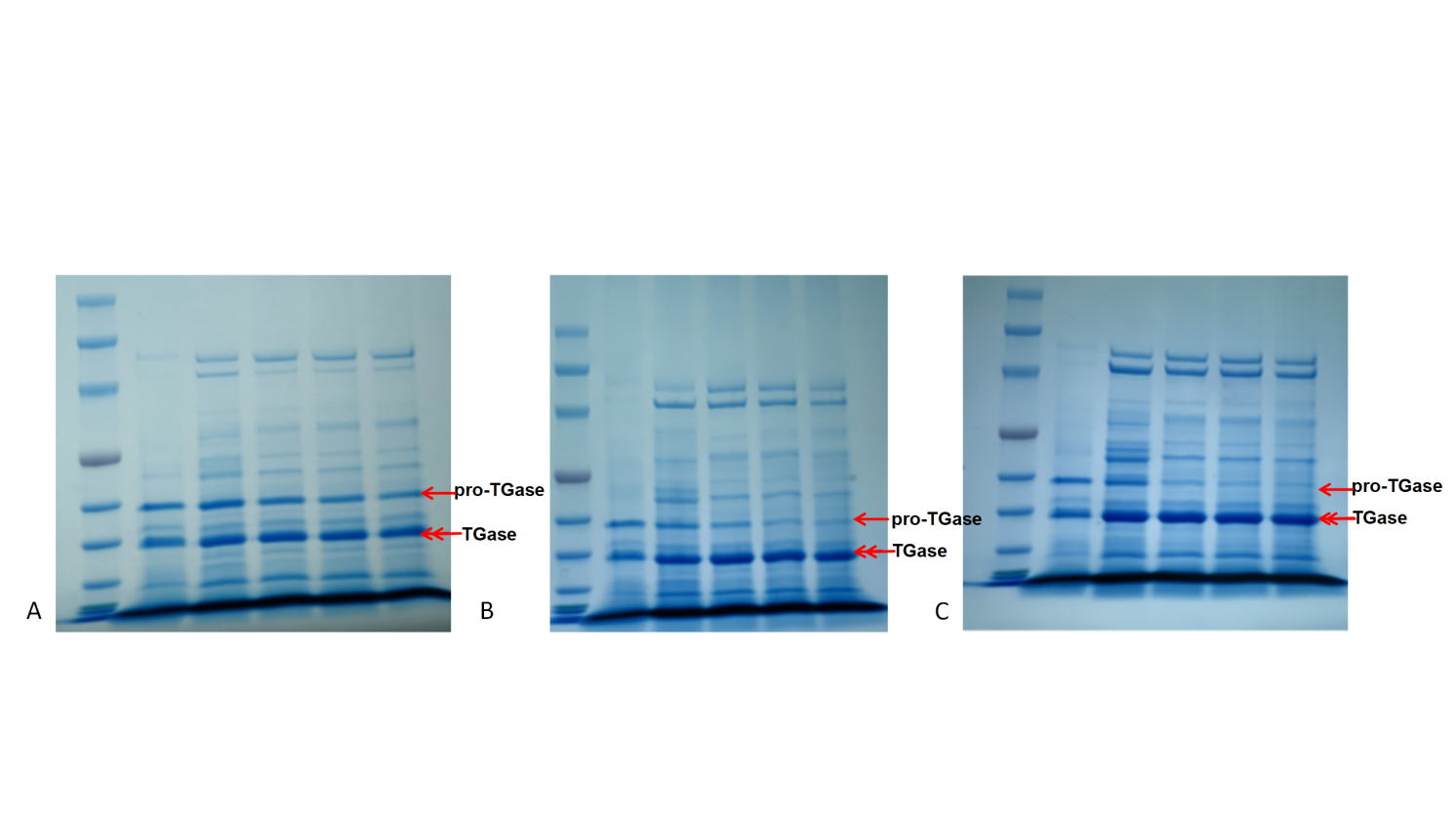
**

**Fig. S5** SDS-PAGE analysis of culture supernatants at different time points during fermentation. Channel M: protein marker. Samples were taken at 12, 24, 36, 48, and 60 hours. (A) Fermentation supernatant of SmDL. (B) Fermentation supernatant of SmGL. (C) Co-culture fermentation supernatant of SmGL and *B. amyloliquefaciens* CICC10888.

**
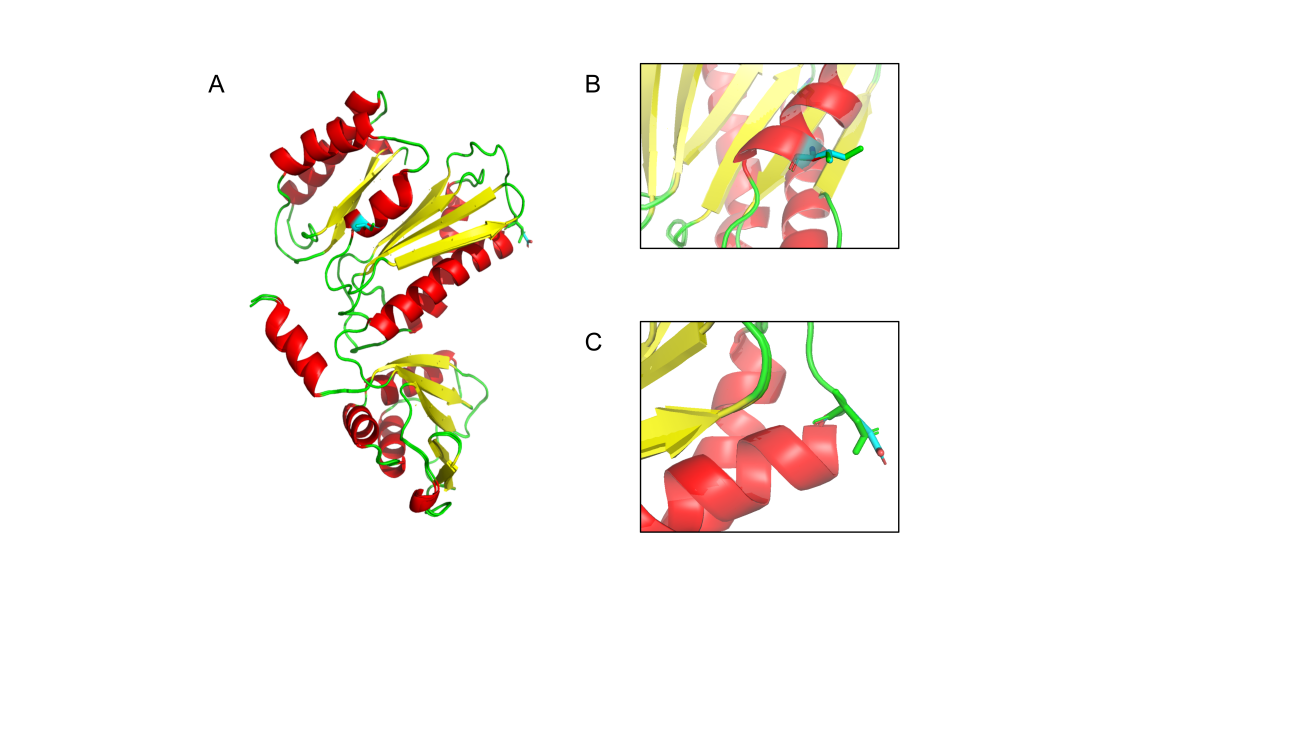
**

**Fig. S6** Aphla fold modeling of the three-dimensional spatial structure of ArgJ protein: A Overlapping forms of the structure of ArgJ protein before and after mutation B Local enlargement of the structure of 79 mutation points C Local enlargement of the structure of 172 mutation points

**Fig. S7** qRT-PCR verification


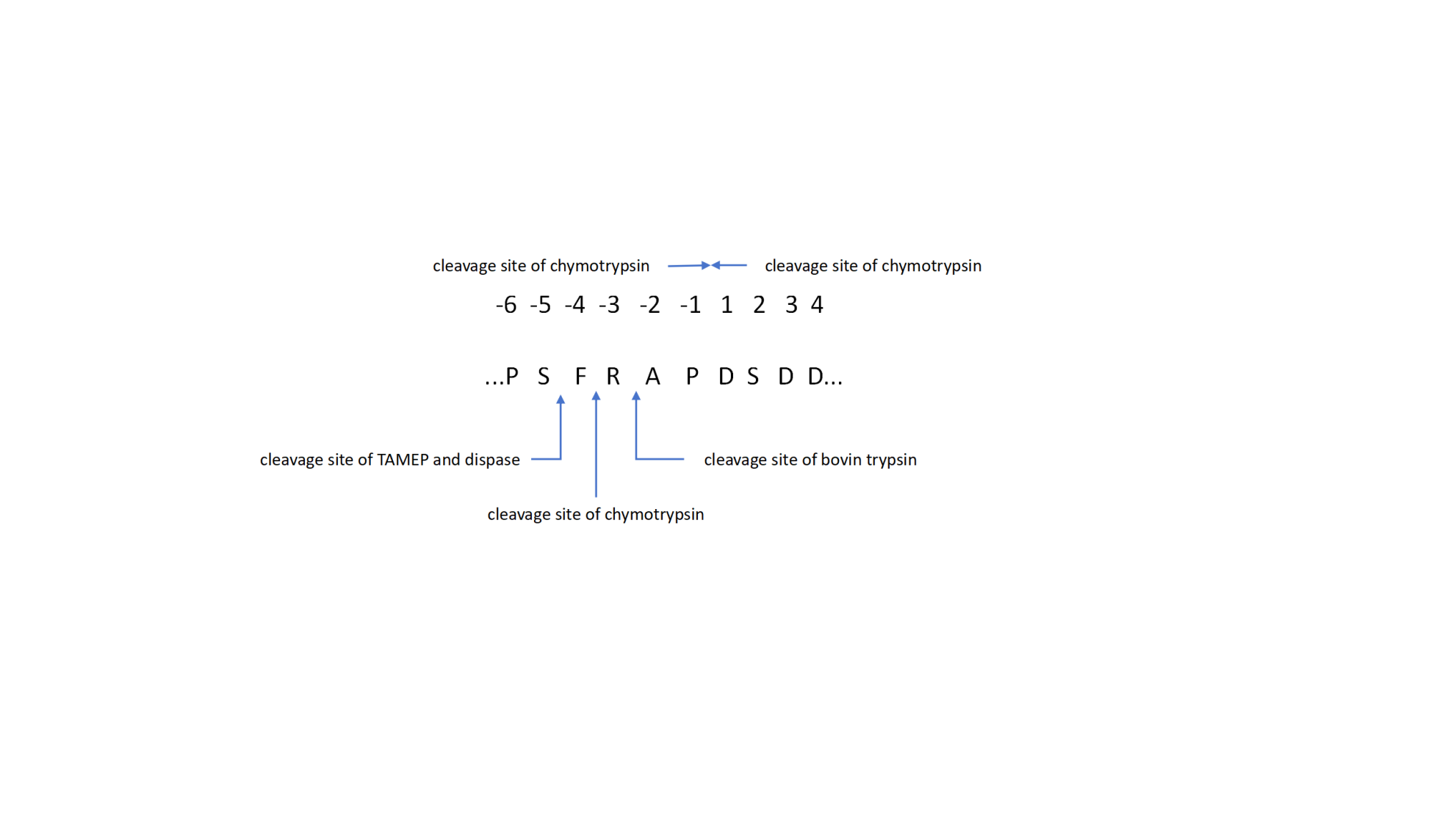


**Fig. S8** Cleavage site of TAMEP, dispase, chymotrypsin and bovine trypsin to pro-mTGase

**Fig. S9** Activation efficiency of pro-TGase by different kinds of proteases

# Supplementary Data

**S3.9** **Effect of Inoculum ratio and time on TGase production**

The quantity of inoculum plays a pivotal role in the process of fermentation, particularly in co-culture systems. Inadequate inoculation of one strain may result in its weakened growth and hinder co-cultivation. As depicted in Fig. 6A, when the inoculum ratio for both strains was set at 10% (v/v), the TGase activity in *B. amyloliquefaciens* CICC10888, which underwent a 100-fold dilution for inoculation, reached its peak at 8.73 U/mL.

The co-culture exerted a significant influence, as illustrated by the optimized results in Fig. 6B. The peak TGase activity (10.13 U/mL) was achieved 4 hours after inoculation with SmGL. Consequently, the optimal timing for inoculating B. amyloliquefaciens CICC10888 was determined to be 4 hours post-inoculation with SmGL.
